# Exploring Metabolic Mechanisms in Calcific Tendinopathy and Shoulder Arthrofibrosis: Insights and Therapeutic Implications

**DOI:** 10.3390/jcm13226641

**Published:** 2024-11-05

**Authors:** Shahenvaz Alam, Marisa Shauna Sargeant, Ronak Patel, Prathap Jayaram

**Affiliations:** Department of Orthopedics, Musculoskeletal Institute, School of Medicine, Emory University, Atlanta, GA 30329, USAmarisa.shauna.sargeant@emory.edu (M.S.S.); ronak.patel@emory.edu (R.P.)

**Keywords:** rotator cuff, adhesive capsulitis, calcium homeostasis, fibrosis, inflammation

## Abstract

Rotator cuff calcific tendinopathy and arthrofibrosis of the shoulder (adhesive capsulitis) are debilitating musculoskeletal disorders that significantly impact joint function and impair quality of life. Despite its high prevalence and common clinical presentation, the metabolic mechanisms underlying these conditions characterized by pain, and reduced mobility, remain poorly understood. This review aims to elucidate the role of metabolic processes implicated in the pathogenesis of calcific tendinopathy and shoulder arthrofibrosis. We will be focusing on the mechanistic role of how these processes contribute to disease progression and can direct potential therapeutic targets. Calcific tendinopathy is marked by aberrant calcium deposition within tendons, influenced by disrupted calcium and phosphate homeostasis, and altered cellular responses. Key molecular pathways, including bone morphogenetic proteins (BMPs), Wnt signaling, and transforming growth factor-beta (TGF-β), play crucial roles in the pathophysiology of calcification, calcium imbalance, and muscle fibrosis. In contrast, shoulder arthrofibrosis involves excessive collagen deposition and fibrosis within the shoulder joint capsule, driven by metabolic dysregulation and inflammation. The TGF-β signaling pathway and inflammatory cytokines, such as interleukin-6 (IL-6), are central to the fibrotic response. A comparative analysis reveals both shared and distinct metabolic pathways between these conditions, highlighting the interplay between inflammation, cellular metabolism, extracellular matrix remodeling, calcific deposition, and calcium migration to the glenohumeral joints, resulting in adhesive capsulitis, thereby providing insights into their pathophysiology. This review discusses current therapeutic approaches and their limitations, advocating for the development of targeted therapies that address specific metabolic dysregulations. Future therapeutic strategies focus on developing targeted interventions that address the underlying metabolic dysregulation, aiming to improve patient outcomes and advance clinical management. This review offers a comprehensive overview of the metabolic mechanisms involved in calcific tendinopathy and shoulder arthrofibrosis, providing a foundation for future research and therapeutic development.

## 1. Introduction

Rotator cuff calcific tendinopathy (RCCT) disease is a condition characterized by inflammation or degeneration of the tendons in the rotator cuff, often resulting in limited range of motion (ROM) and severe pain. In most cases, it may also involve calcific deposition, where calcium builds up within the tendons of the rotator cuff [1].

Similarly, a condition known as shoulder arthrofibrosis, widely termed adhesive capsulitis (AC), can mimic the symptoms of rotator cuff calcific tendinopathy. The AC is a pathologic shoulder condition characterized by progressive glenohumeral joint stiffness and restricted range of motion with considerable pain [2,3,4,5,6,7,8,9,10,11,12,13,14]. Hence, it is often called a “Frozen shoulder”. It can manifest as primary adhesive capsulitis, which arises idiopathically without a discernible etiology, or as secondary adhesive capsulitis, which develops after events such as surgical interventions or trauma to the shoulder [15,16,17]. This condition is characterized by nocturnal pain that significantly impairs daily activities. Generally, it is defined by a loss of range of motion (ROM) greater than 25% in at least two planes and passive external rotation loss that is greater than 50% of the uninvolved shoulder or less than 30° of external rotation [18]. The estimated prevalence of adhesive capsulitis ranges from 2% to 5% within the general population [19]. The precise etiology of the disorder remains elusive, primarily due to the complexity of its inflammatory pathogenesis. The overlap in symptomatology among these disorders frequently presents diagnostic challenges for medical professionals.

To this end, calcium homeostasis within skeletal muscle cells plays a pivotal role in preserving the structural integrity and functionality of tissue. Elucidating the precise mechanisms behind calcium homeostasis as it pertains to shoulder pathology will certainly give insight to the underlying disorder.

The purpose of this review is to elucidate the possible mechanisms underlying rotator cuff diseases like calcific tendinopathy, rotator cuff tears (RCT), and AC, with a particular focus on calcium homeostasis. The review delves into how calcific interventions contribute to the advancement of arthrofibrosis and calcific tendinopathy, along with their associated comorbidities. We aim to underscore the metabolic mechanism, which is amplified by cell-mediated pathways, contributing to the progression of rotator cuff diseases. Furthermore, it will highlight strategies for prevention, management, and therapeutic measures to be undertaken following prognosis.

## 2. Background and Etiology

Rotator cuff calcific tendinopathy (RCCT) is a condition involving the formation of calcium deposits within the tendons of the rotator cuff, which is a group of muscles and tendons that stabilize the shoulder joint. While it can impact any tendon in the rotator cuff, it frequently affects the supraspinatus tendon (with an incidence of 51.5–90% of cases) [20]. Calcifications in the supraspinatus tendon were found to be significantly associated with pain. Symptoms may be linked to muscle spasms, inflammation of the subacromial bursa (bursitis), issues with the long head of the biceps tendon, secondary adhesive capsulitis, or rotator cuff tears (RCT) [21,22]. The common notion prevalent here is the ubiquitous calcific depositions in all the associated disorders. Differentiating between RCCT, AC, and RCT is clinically challenging due to their overlapping presentations of shoulder pain and stiffness. All three pathologies involve the shoulder tendons and are crucial differential diagnoses in patients exhibiting shoulder discomfort [23]. However, the etiologies of these conditions differ considerably.

RCCT is characterized by the deposition of calcium hydroxyapatite crystals within the rotator cuff tendons. Adhesive capsulitis results from prolonged immobility, leading to capsular contracture and fibrosis, often after trauma or postoperative immobilization. These conditions cause discomfort and limit daily activities, leading to reduced quality of life. AC most commonly develops in individuals between the ages of 40 and 60, with occurrences in patients over 70 being rare except in cases of secondary traumatic adhesive capsulitis [9]. The incidence rate in women is 1.6 to 4 times higher than in men [24]. Additionally, there is a higher prevalence of AC among African American and Hispanic/Latino populations [11]. In certain individuals, chronic pain from calcific tendinitis can lead to reduced use of the shoulder, potentially resulting in secondary adhesive capsulitis [25]. RCT involves repetitive microtrauma to the rotator cuff tendons, frequently accompanied by inflammation of the subacromial bursae, which normally facilitate joint lubrication. The deposition of calcium can lead to calcific bursitis, which can sometimes develop due to prolonged inflammation [26]. Calcific tendinopathy may ultimately develop into bursitis and inflammatory synovitis as a result of chemical irritation from the calcific deposits. This irritation leads to swelling and elevated local pressure in the tissues, creating chemical furuncles. As the bursa thickens, it causes compression in the subacromial space, resulting in extreme shoulder pain [27]. In approximately 25% of patients with calcific tendinitis, a concurrent rotator cuff tear is observed. Notably, these tears are more frequently associated with smaller calcific deposits than larger ones [27]. The shoulder pain and stiffness also occur when there is a chronic calcium migration into the subacromial–subdeltoid bursa. In rare conditions, the intramuscular migration of calcific deposits was shown by the research team where they highlighted the use of ultrasound for the diagnosis of calcific tendinopathy and extreme cases of calcium migration into the surrounding tissues, including the subacromial–subdeltoid bursa and medially to the myotendinous junction of the subscapularis [28]. However, the primary noteworthy complication in the natural progression of calcific tendinopathy is a phase of adhesive capsulitis. Fortunately, this condition is always reversible, and calcific deposits are replaced by healthy tissue [29].

Routine diagnosis of shoulder conditions using ultrasound/radiological survey provides an effective strategy as most of the cases are asymptomatic during the initial phase. Treatment choices for calcific deposits range from conservative measures like rest, physical therapy, nonsteroidal anti-inflammatory drugs, and shock wave therapy to surgical options such as arthroscopic removal of the calcified area [29]. The utilization of non-invasive or minimally invasive techniques for these shoulder conditions paves the way for innovative treatment options. Recently, the technique utilizing ultrasound-guided treatment options for both calcific tendinopathy and AC has been proposed. “The Rizzoli Technique” as it was called by the researchers suggested a minimally invasive ultrasound-guided treatment with a 12–5 Mhz linear probe for calcific deposits in supraspinatus or infraspinatus tendons and other locations in subscapularis tendon. Additionally, the saline solution was injected with pressure to distend the capsule for AC treatment. Finally, one cc of Methylprednisolone was injected into the subdeltoid bursa and glenohumeral joint for the rehabilitation of the patients with calcific tendinopathy and AC [30].

The functional implications of these disorders are profound. Patients often report difficulty performing routine tasks such as reaching overhead, lifting objects, or even sleeping comfortably. This functional limitation can result in significant psychological distress, including anxiety and depression. As such, effective management strategies are essential for alleviating symptoms and restoring functionality.

**Figure 1:**
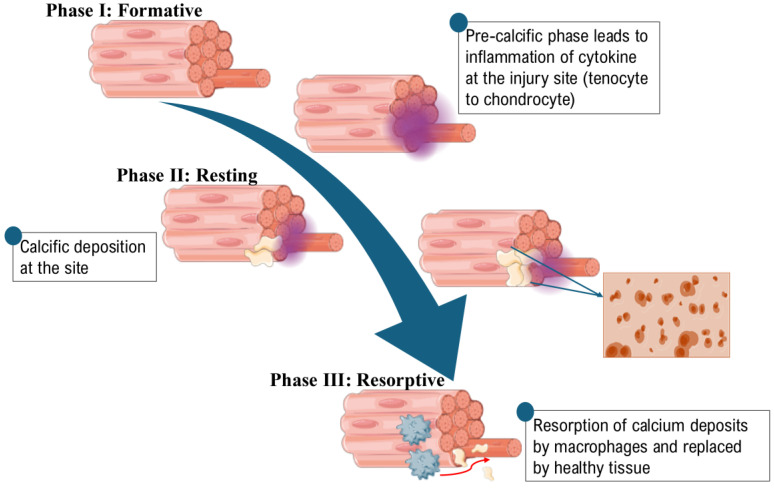
The three phases of calcific deposition in rotator cuff due to injury/inflammation; Phase I mainly shows pre-calcific phase where inflammation leads to recruitment of pro-inflammatory cytokines and replacement of tenocytes to chondrocytes; Phase II is referred to as the resting phase, during which calcific depositions form at the injury site, leading to a cessation of inflammation from Phase I. In Phase III, the presence of severe pain characterizes the phase, but eventually, the pain subsides as calcific deposits are resorbed. This process occurs through cell-mediated phagocytosis facilitated by macrophages, followed by the replacement of damaged tissue with healthy tissue. The figure was created using Servier Medical Art, licensed under a Creative Commons Attribution 4.0 License. https://creativecommons.org/licenses/by/4.0/ (accessed on 21 October 2024).

However, the progression of calcific tendinopathy of the rotator cuff has diverse processes as shown in Figure 1. The calcific stage comprises three sequential phases: formative, resting, and resorptive. Preceding calcification, during the pre-calcific stage, tenocytes undergo metaplasia into chondrocytes, prompting a fibrocartilaginous transformation within the tendon. In the formative phase of the calcific stage, calcium deposits initiate and progressively enlarge. This process ceases during the resting phase. In the subsequent resorptive phase, which is a very painful condition, calcific deposits are degraded and absorbed via cell-mediated phagocytosis, executed by macrophages, polymorphonuclear cells, and fibroblasts [25,27]. After calcium resorption, patients enter the post-calcification stage, where the tendon starts to heal. During this stage, the fibers realign, and the calcium deposits are eliminated [31]. Calcium dysregulation or the failed healing process of tendons contributes to the calcific progression and leads to acute shoulder pain and AC. Moreover, calcific deposits in tendinopathy and the articular infiltration of calcium at the synovial/capsular joint and inflammation can be associated with these two shoulder disorders as suggested by ultrasound/sonographic findings [32]. However, the underlying mechanisms remain insufficiently explored, and there is no scientific evidence to elucidate their etiology [1].

Likewise, AC is characterized and classified into primary and secondary forms. Primary (idiopathic) adhesive capsulitis arises spontaneously without any apparent cause or precipitating trauma. This type occurs independently, with no specific incident or injury leading to the onset of symptoms, making it more difficult to predict and manage due to its uncertain etiology. Secondary adhesive capsulitis develops because of an identifiable cause, such as trauma, surgical procedures, or underlying medical conditions like diabetes or thyroid disorders. In these cases, the condition emerges as a response to the specific injury or health issue [8,33].

**Figure 2:**
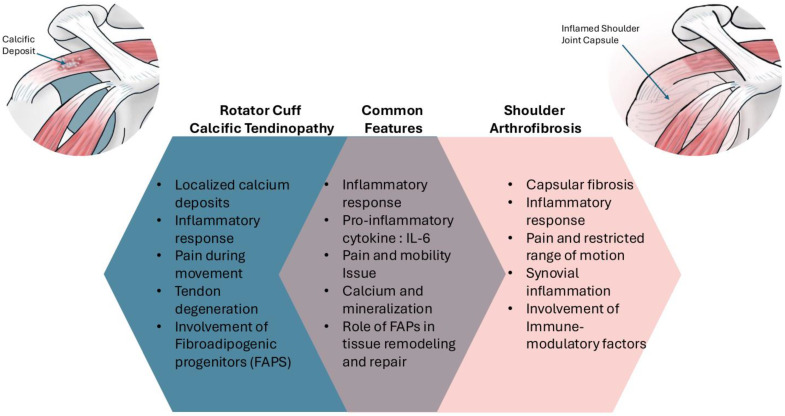
Summary of the similarities and potential differences between rotator cuff calcific tendinopathy and shoulder arthrofibrosis (adhesive capsulitis).

Investigating the metabolic mechanisms underlying calcific tendinopathy and shoulder arthrofibrosis is vital for several reasons (Figure 2). Metabolic pathways play a crucial role in the development and progression of these conditions. Disruptions in normal metabolic processes can lead to pathological changes in the tendons and joint capsules, contributing to the clinical manifestations of pain and immobility. Understanding these pathways can reveal potential therapeutic targets that may provide more effective treatments than current options. Table 1 highlights the pathophysiological link between calcific tendinopathy and shoulder arthrofibrosis (adhesive capsulitis).

Moreover, existing literature highlights several knowledge gaps regarding the specific metabolic alterations present in these disorders. For instance, while inflammation is a well-documented factor, the precise role of metabolic dysregulation, such as alterations in calcium and phosphate homeostasis or cellular energy metabolism, diverse cell fate commitment, and the proliferation of tenocytes remains poorly understood. Addressing these gaps through focused research can significantly advance our understanding and treatment of rotator cuff disorders.

## 3. Calcium as a Vital Signaling Molecule During Muscle Contraction

**Figure 3:**
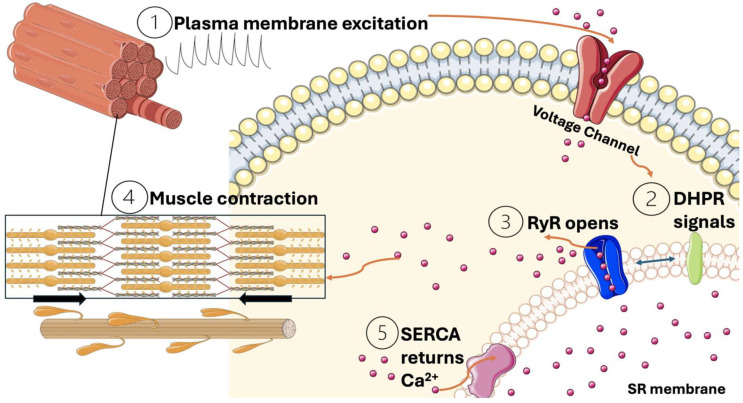
Illustration of common contraction mechanism in skeletal muscle where the following processes occur: 1. transmission of impulses through voltage channels results in the activation of DHPR receptors; 2. DHPR receptors in sarcolemma tubules make conformational changes and transmit the impulses to the Ryanodine receptor (RyR); 3. RyR opens the calcium ion influx from the sarcoplasmic membrane to the skeletal muscle; 4. calcium ions initiate muscle contraction; and 5. during the end of muscle contraction, calcium ions return to the sarcoplasmic membrane due to the Ca^2+^-ATPase pump (SERCA). The figure was created using Servier Medical Art, licensed under a Creative Commons Attribution 4.0 License. https://creativecommons.org/licenses/by/4.0/ (accessed on 22 October 2024).

Calcium ions (Ca^2+^) are crucial signal molecules in the process of muscle contraction. The excitation coupling contraction, also known as ECC, is activated with fast sodium channels (postsynaptic voltage channels, SCN4A) [34]. An action potential travels along the sarcolemma and into the T-tubules, inducing a conformational change in the voltage-sensitive dihydropyridine receptors (DHPR). This change prompts the opening of ryanodine receptors (RyR) on the sarcoplasmic reticulum, causing a rapid efflux of Ca^2+^ into the cytosol [35]. The resulting increase in intracellular calcium concentration allows Ca^2+^ to bind to troponin C, a regulatory protein within the troponin complex on the actin filament. This binding causes tropomyosin to shift, exposing the myosin-binding sites on the actin filaments. As a result, myosin heads can attach to actin, forming cross-bridges and initiating an ATP-dependent cycling process that produces contractile force. Contraction ends when Ca^2+^ is pumped back into the sarcoplasmic reticulum by the Ca^2+^-ATPase pump (SERCA), returning the muscle fibers to a relaxed state as depicted in Figure 3 [36,37].

Calcium’s versatility as a secondary messenger is due to its interactions with various proteins and enzymes, leading to, for example, changes in transcription, enzyme activity modulation, and alterations in cellular structures [38]. The role of Ca^2+^ is tightly regulated to ensure precise cellular responses, maintaining cellular homeostasis and function.

## 4. Calcium’s Role in Tendon Stem Cell Modulation and Proliferation

The mechanism of calcific tendinopathy remains controversial, as its exact etiology continues to be undefined. During the resorptive stage of the disease, symptoms are most severe due to the softness and instability of calcium deposits. Calcium can extend beyond the typical resorption areas and become deposited in surrounding tissues at this stage [39]. Common targets for migration include the bursae, bone (intraosseous migration), muscles, and occasionally other nearby tissues. Various hypotheses have been proposed to explain the pathological processes involved, including chronic tissue degeneration due to wear and tear [39], cumulative damage from recurring minor injuries, tenocyte apoptosis, inflammatory or immune responses leading to calcification, and endochondral ossification which followed a process akin to bone formation where calcified deposits replace cartilage [40]. Nevertheless, these hypotheses have not been entirely satisfactory in explaining all occurrences of calcific tendinopathy. Extrinsic factors like age and body mass index (BMI) have been strongly linked to shoulder pain. The loss of muscle mass, prolonged inactivity, and sudden exposure to mechanical stress turned out to be aggravating the more serious complications. Moreover, the rise in shoulder pain and tears is similar to conditions such as shoulder arthrofibrosis and rotator cuff tears, making the understanding of this issue more complex [41].

The muscle tissues are sensitive to mechanical stress and are mainly controlled by the diverse calcium-related pathways that lead to diverse changes in the developmental program [42]. Recent studies and evidence suggest a potential mechanism through which cell-mediated pathways contribute to calcification and the progression of fibrosis. These new insights may help elucidate the intricate relationships and interactions between calcium and various metabolic pathways in fibrosis. Studies on tendon stem cells (TSCs) within the specialized microenvironments, known as niches, have uncovered novel insights into the underlying disease mechanisms [1,25]. These niches represent a three-dimensional specialized milieu that is crucial for maintaining a balance between self-renewal and cell-fate determination. The findings indicate that the TSC niche is predominantly constituted by the extracellular matrix (ECM), with biglycan, proteoglycan, and fibromodulin playing pivotal roles in its structural organization [43,44]. Moreover, the pathological process is likely driven by cell-mediated mechanisms rather than solely by the accumulation of inorganic calcium hydroxyapatite, involving the transformation of tenocytes into chondrocytes through fibrocartilaginous metaplasia, potentially due to tissue hypoxia [45,46]. More research corroborates that under conditions of excessive loading and repetitive microtrauma, TSCs may undergo aberrant differentiation [47]. This aligns with the theory of failed cell-mediated healing and leads to the progression of calcific tendinopathy [48,49].

The excess of calcium and tendon biology share a correlation as Passini et al. suggested that the Ca^2+^ signals observed in their experiments could activate and upregulate enzymatic collagen cross-linking, which in turn may lead to increased tendon stiffness and strength, ultimately affecting physical performance [50]. The mechanical stress on the tendons has a discrete effect and is involved in the myriads of signaling mechanisms. For instance, the slight shear on the tendons upregulated the genes associated with tendon preservations such as SCX and TNMD [51,52], enhanced collagen production and increased cell death, glycosaminoglycan content, and collagenase activity [52,53,54]. However, the increase in mechanical stress has adverse effects on the tendon cells. They maintain cellular integrity but increase MMP3, MMP13, MMP9, and collagen production and synthesis [55]. In more detrimental cases, it elevated the expression of COL1A1, IL-6 with massive collagen bundle rupture and abnormal nuclear morphology [56,57,58,59]. Restructuring the extracellular matrix during the progression of tenocytes under disease conditions results in the formation of dense collagen, a characteristic hallmark of AC. Moreover, the overexpression of MMP9 and IL-6 at the joint capsule has been observed in the rat model, suggesting a role in AC [8,60]. Thus, the presence of aberrant immunological or fibroblast proliferation due to dysregulation of certain factors such as calcium may significantly enhance the pathophysiology of tendinopathy and AC.

## 5. Modulation of Calcium Channels

Tenocytes detect mechanical forces through the mechanosensitive ion channel Piezo1, which responds to the shear stresses generated by the sliding of collagen fibers [61]. The Piezo1 channel has gained a lot of attention due to its role in Ca^2+^ influx-mediated signaling pathways and pathological disorders such as skeletal, neurological, pulmonary, and cardiovascular disorders as well as cancers [62]. The experiment conducted on human smooth muscles demonstrated that the upregulation of Piezo1 contributes to the osteogenic responses following cell calcification [63]. The sensitivity of Piezo1 membrane ion channels to Ca^2+^ offers a promising therapeutic intervention. Utilizing several pharmacological drugs as antagonists to these channels holds significant potential. The targeting of the Piezo1 channel using non-specific and specific inhibitors involves three primary molecular mechanisms. These mechanisms encompass the direct blockade of the channel’s ion-conducting pore (Gd^3+^, ruthenium red), modulation of the proximal lipid bilayers (GsMTx4, Aβ peptides, fatty acids), and competitive inhibition (Dooku1, tubeimoside I, salvianolic acid B, jatrorrhizine, escin) with the Piezo1 activator Yoda1 in its binding sites. Consequently, Piezo1 inhibitors have the potential to suppress the entry of Ca^2+^ ions into the intracellular space, thereby influencing a network of intricate signaling pathways that are pivotal in numerous physiological and pathological processes [64]. Interestingly, salvianolic acid B was utilized as a therapeutic intervention in AC, attenuating inflammation and decreasing fibroblast proliferation [65,66]. Hence, the mishandling of Ca^2+^ signals play an important role in TSCs and tendon progenitor cells during calcific progression and could be linked with the cause of undefined idiopathic AC.

Another research finding identified the dynamic expression of endogenous Ca_V_1.2, the L-type voltage-dependent Ca^2+^ channel in tendon fibroblasts during tendon development and homeostasis. In the study, the gain of function mutation in Ca_V_1.2 promotes tendon formation and the proliferation of tendon fibroblast [67,68,69]. Notably, upregulation of Ca^2+^ significantly increased collagen fibrillogenesis which leads to a condition of tendon hypertrophy. While there are several Ca_V_1.2 agonists like BayK-8644 or FPL 64176 that can mimic the effect of Ca_V_1.2 by increasing the influx of Ca^2+^ across the plasma membrane, finding antagonists for these channels could potentially address associated disorders [70].

## 6. Calcium Homeostasis in Skeletal Muscle Fibro-Adipogenic Progenitor Cells

Fibro-adipogenic progenitors (FAPs) are muscle-resident interstitial cells exhibiting mesenchymal stem/stromal cell properties, characterized by the expression of markers such as PDGFRα, CD90, and SCA-1 [71]. They are pivotal for muscle homeostasis and regeneration, actively shaping the microenvironment through the secretion of extracellular matrix components, cytokines, and immune-modulatory factors [72,73]. Following muscle injury, FAPs differentiate into activated fibroblasts, adipocytes, and osteogenic cells [74,75,76], thereby providing essential signals for muscle stem cell (MuSC) expansion and myogenesis. Calcium overload and myofiber disruption leads to FAP pathogenicity. The repeated overload of calcium leads to fibrosis and adipogenesis [73,77,78]. The dysregulation of FAP functions and TGF-β signaling pathways—where TGF-β cytokines like TGF-β1, -β2, and -β3 activate SMAD2 and SMAD3 in canonical pathways, as well as non-canonical pathways like PI3K-AKT and p38 MAPK—can result in excessive fibrosis and impaired muscle regeneration. There are reports of the activation of IL-6 inflammatory cytokines through the PI3K-AKT pathway in synovial fibroblast and the progression of fibrosis in AC [79]. Additionally, myostatin influences FAP activation via p38 MAPK and AKT pathways. Myostatin is also known as GDF8 (growth differentiation factor 8), belongs to TGF-β1, and is a factor for differentiation and muscle mass growth. Calcium signaling plays a crucial role in its responsiveness [80,81]. Myostatin treatment has been associated with various conditions such as muscular disorders, diabetes, hypertension, and hyperlipidemia [82,83]. However, the interconnected mechanisms require careful consideration, as they can affect other related pathways and cause side effects during clinical trials. Understanding these complex mechanisms is crucial for developing targeted therapies to enhance muscle regeneration and reduce fibrosis in various muscular disorders. Additionally, it is important to understand the relationship between calcium and its effects on fibrotic disorders, as this knowledge is crucial for elucidating the signaling pathway of TGF.

## 7. Calcium as an Activator of Signaling Cascade

Calcium regulation is crucial for modulating TGF-β signaling, as calmodulin binds to SMAD2, SMAD3, and SMAD4 in the presence of Ca^2+^. Calcium and calmodulin play vital roles in cellular signaling and regulation. When intracellular calcium levels rise, calmodulin binds to Ca^2+^, undergoing a conformational change that enables it to interact with and modulate various target proteins, including kinases and phosphatases. This interaction influences numerous physiological processes, such as muscle contraction, neurotransmitter release, and cell growth. The overexpression of calmodulin can reduce TGF-β’s effects on 3TP-Lux activity (transcriptional activity driven by the 3TP [three TGF-β-responsive elements] promoter), [84] by inhibiting SMAD2 through specific interactions in its Mad homology (MH)1 domain (key domain in SMAD’s). Additionally, Ca^2+^-calmodulin-dependent protein kinase II (CaM kinase II) may phosphorylate SMAD2 and SMAD4, further inhibiting their activity [85]. Moreover, TGF-β-induced increases in intracellular calcium concentration (Ca^2+^) occur through the release of calcium from internal stores and the influx from the extracellular space via plasma membrane channels. This influx is mediated by inositol 1,4,5-trisphosphate (IP3)-gated channels after phospholipase C activation. IP3 binding to its receptor triggers calcium release into the cytosol, followed by secondary influx via store-operated channels. TGF-β treatment has been shown to phosphorylate type I IP3R in mesangial cells. Ca^2+^ also influences the actin cytoskeleton and cell motility, and its influx leads to significant crosstalk with the extracellular signal-regulated kinase (ERK) pathway, highlighting its role in TGF-β signaling modulation [86,87].

The activated Ca^2+^·calmodulin (CaM) complex regulates metabolic processes by influencing enzymes such as phosphodiesterases and protein kinases. While calmodulin itself lacks enzymatic activity, its activation enables it to bind to specific short peptide sequences in target proteins, leading to structural changes that enhance its functional activity. In the cAMP-protein kinase A (PKA) pathway, elevated calcium concentrations can amplify PKA’s effects, thereby impacting metabolism and FAP differentiation [88,89,90,91]. Recent studies have also indicated that the Wnt-Ca^2+^ signaling pathway may be involved in altered adipocyte cellularity [92]. Specifically, the binding of Wnt proteins to frizzled (Fzd) receptors activates G proteins and phospholipase C (PLC), resulting in increased intracellular calcium levels [93]. This, in turn, activates protein kinase C (PKC) and Ca^2+^/calmodulin-dependent protein kinase II (CaMKII), further influencing metabolic pathways [88,93]. The Ca^2+^/CaMKII-related pathways are often associated with the progression of diabetes, a well-known comorbidity with AC syndrome, and could be associated with its development [94]. The complications of calcification and AC are linked to other diseases such as hyperthyroidism and other hormonal diseases. The involvement of calcium in these disorders should not be neglected, as the underlying mechanism suggests hyperthyroidism enhances the production of calcium in serum levels and leads to hypercalcemia. Although there are very few cases of hyperthyroidism associated with AC and other tendinopathy, hypothyroidism was found to be a major player in the disease progression [95]. However, the mechanistic role is still undefined. Likewise, parathyroid hormone (PTH) and calcitonin play crucial roles in regulating calcium homeostasis. PTH is secreted by the parathyroid glands and functions to elevate plasma calcium levels by stimulating osteoclast activity, which breaks down bone tissue to release calcium, enhancing renal calcium reabsorption, and indirectly increasing intestinal calcium absorption through the activation of vitamin D [96]. Conversely, calcitonin, a hormone produced by the parafollicular cells of the thyroid gland, lowers plasma calcium levels by inhibiting osteoclast activity, thereby reducing bone resorption and potentially promoting calcium excretion by the kidneys. Together, these hormones maintain a balanced calcium concentration in the blood [97]. Henceforth, calcitonin as an intranasal spray has been considered as a treatment option in AC [98]. Moreover, salmon calcitonin showed an effective treatment in alleviating the pro-inflammatory cytokines related to fibrosis in synovial/capsular fibroblast [99]. Similar to PTH and calcitonin, other hormonal regulation affects musculoskeletal disorders. For instance, the hormonal imbalance in females significantly comes out as a risk factor for rotator cuff tendinopathy and AC. Estrogens and progesterone promote fibroblast proliferation and type I collagen production in the musculoskeletal system. However, menopause leads to a notable decrease in type I collagen in tendons, likely due to reduced hormone levels and higher levels of pro-inflammatory cytokines (IL-6, TNF-α), common postmenopausal changes [100]. Researchers suggest that genetic predisposition to fibrosis and hormones like estrogen and thyroid-stimulating hormone may contribute to AC [101]. This condition affects about 2% to 5% of people, mainly middle-aged women and often the nondominant arm. A preliminary study identified that hormonal replacement therapy (HRT) could be an effective therapy against AC in menopausal women [102]. The interaction between ER-α and ER-β, the predominant isoforms of estrogen receptors, results in varying effects on tissues due to their complex interplay. In the mammary gland, 17β-estradiol influences specific signaling pathways depending on the receptor isoform it binds to; activation of ER-α stimulates cellular proliferation, while activation of ER-β inhibits proliferation and promotes differentiation. Consequently, differential expression levels of these receptors can elicit opposing biological responses within the same tissue. Further, the overexpression of ER-β has been identified in the tenocytes and synovial tissues of postmenopausal individuals, suggesting a potential role of estrogen receptors in the disease’s pathogenesis [100,103]. The gain of insights with this research paves the way for a better understanding of the progression as well as the correlation of calcium imbalance among different rotator cuff diseases.

Dissecting through the mechanism of calcium and its role in metabolic pathways, the effects of calcium on mesenchymal stem cells (MSCs) and its aspect in osteogenic differentiation need a mention here. During the bone remodeling cycle, osteoclast-mediated bone resorption elevates extracellular calcium concentrations to approximately 40 mM, which is critical for regulating osteoblast proliferation, differentiation, and activity [104,105]. The differentiation of human bone-marrow-derived mesenchymal stem cells (MSCs) into osteoblasts is accompanied by the expression of calcium-binding proteins [106]. The complex genetic programs governing osteogenesis are still being elucidated, with RUNX2 identified as a master transcription factor essential for osteoblastic differentiation. Key components of the bone matrix include osteocalcin, collagen, osteonectin, and osteopontin, along with recently identified markers such as CRYab. Other signaling molecules that can originate in bone and have anabolic/hypertrophic effects on skeletal muscle include insulin-like growth factor 1 (IGF1) and bone morphogenetic protein 2 (BMP2), which certainly have a direct connection with skeletal muscle [107]. IGF1 and IGF2 are mitogen factors that are responsible for various fibrotic disorders by enhancing the deposition of ECM after tissue damage [108,109].

Calcium dynamics in MSCs play a significant role in differentiation, with Ca^2+^ and phosphate ions inducing osteogenic differentiation through BMP/SMAD and RAS signaling pathways. SMAD1/5/8 proteins modulate BMP-mediated osteogenesis, and BMP receptors regulate BMP signaling intensity through SMAD1 C-terminal phosphorylation [110]. Additionally, non-SMAD pathways, such as RAS/Raf/ERK, are activated by BMP2 [111]. BMPs, part of the TGF-β family, bind to type I and II serine–threonine kinase receptors, transmitting signals via SMAD and non-SMAD pathways. Recent findings indicate that calcium upregulates BMP2 gene transcription in human MSCs, with elevated BMP2 and BMP6 expression observed in cell culture experiments, highlighting the role of BMP signaling in calcium phosphate-driven osteogenesis [110].

Ca^2+^ and BMP2 have been shown to collaboratively stimulate osteoblast differentiation by enhancing specific osteogenic signaling pathways [112,113]. This synergy is evidenced by the increased phosphorylation of SMAD1/5,6 and GSK3β (glycogen synthase kinase 3), along with elevated β-catenin levels, leading to the significantly higher expression of osteocalcin, RUNX2, and Osterix, ultimately promoting greater bone formation in vivo [113]. Recent studies have highlighted the cooperative crosstalk between Ca^2+^ and BMP2 in osteoblasts, particularly through the induction of the calcium-dependent transcription factor NFATc1 by BMP2. NFAT (nuclear factor of activated T cells) transcription factors are essential for osteoblast differentiation and bone formation [114,115], as they activate osteogenesis via interactions with OSX (Osterix) and the stimulation of Wnt/β-catenin signaling [115,116]. Interestingly, cultures exposed to Ca^2+^ alone for 10 days exhibited significantly enhanced SMAD signaling, correlating with the increased expression of BMP2, BMP4, and Axin2 genes [117]. Additionally, Axin2, a target gene of Wnt/β-catenin signaling downstream of GSK3β, was also expressed in long-term cell cultures [110].

Genes involved in inflammation and bone formation include IL6 [118,119,120], a pro-inflammatory cytokine secreted by osteoblasts that influences the MAPK signaling cascade critical for skeletogenesis, and PTGES, which has been linked to decreased cell proliferation and enhanced osteogenesis [121]. IL1, a heterodimeric cytokine, also plays a role in bone remodeling [122]. The evident release of pro-inflammatory cytokines has been interconnected with rotator cuff diseases and their progression.

**Figure 4:**
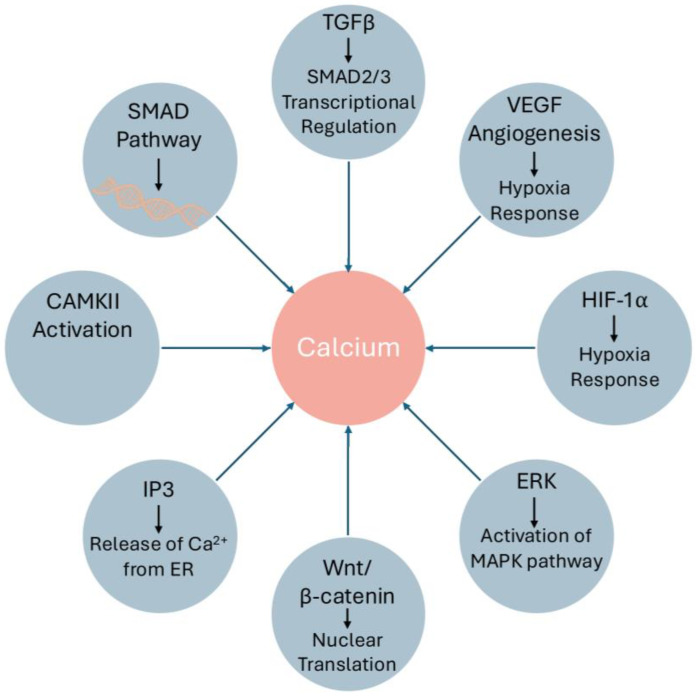
The calcium dynamic is central to various metabolic pathways in the cellular homeostasis and activates certain downstream processes.

Dexamethasone, a synthetic glucocorticoid, promotes the osteoblastic phenotype in bone-marrow-derived osteoprogenitor cells through BMP/SMAD and RAS signaling pathways. The treatment of dexamethasone antagonized remodeling by regulating the TGF-β1/SMAD3 signaling pathway, which is likely to play a role in the treatment of inflammatory diseases [123]. While osteogenic differentiation has mild effects on muscle cells and fibrosis, amorphous calcium carbonate has been shown to enhance the differentiation of primary human muscle cells into myotubes, with excessive calcium deposition leading to osteocyte differentiation when MSCs are treated with amorphous calcium carbonate [124]. Calcium mishandling or delayed calcium signaling suggested the reinforcement of a series of cascades for osteogenic differentiation from multiple inputs and edges to more complex calcific and fibrotic disorders (Figure 4). An in-depth investigation into the molecular mechanisms governing intracellular Ca^2+^ dynamics, and their relationship with the secretion of bone matrix and tissue development described in Table 2, could potentially reveal novel therapeutic avenues for the treatment and management of various bone diseases, including conditions such as adhesive capsulitis and calcific tendinopathy, where aberrant calcium deposition and regulation play a critical role.

## 8. Calcium Homeostasis Under Hypoxic Conditions

Lifestyle diseases, such as obesity, type 2 diabetes, heart disease, and certain cancers, are often linked to poor dietary habits and insufficient physical activity [125]. A sedentary lifestyle can lead to chronic ailments and surgeries, which in turn can result in decreased mobility and increased lethargy. When physical activity is minimal, skeletal muscles receive less oxygen, a condition known as hypoxia. This low-oxygen state activates certain biological responses in the body. Under hypoxic conditions, hypoxia-inducible factors (HIFs) are triggered, allowing cells to adapt to the lack of oxygen. These factors influence various physiological processes like metabolism and the formation of new blood vessels. However, chronic hypoxia can also exacerbate lifestyle-related disorders.

Hypoxia-Inducible Factor 1-alpha (HIF-1α) is a transcription factor that is stabilized and becomes active under low oxygen conditions [126]. In normal oxygen levels, HIF-1α is rapidly degraded. However, under hypoxia, it accumulates and translocates to the nucleus where it dimerizes with HIF-1β. This complex then binds to hypoxia-responsive elements (HREs) on DNA, initiating the transcription of various genes that help the cell adapt to hypoxia. Vascular Endothelial Growth Factor (VEGF) is one of the many genes upregulated by HIF-1α. VEGF is a signal protein that stimulates the formation of new blood vessels, a process known as angiogenesis. This is particularly important under hypoxic conditions as new blood vessels increase the supply of oxygen and nutrients to tissues that are experiencing low oxygen levels [127].

These responses are part of a larger adaptive mechanism by which cells and tissues attempt to cope with reduced oxygen availability, ensuring survival and maintenance of essential functions.

Calcium plays a significant role in the regulation of hypoxia-induced factor (HIF), particularly HIF-1α, in various ways. Changes in intracellular calcium levels can activate several signaling pathways that influence the stability and activity of HIF-1α. For instance, calcium can activate protein kinases such as Calcium/Calmodulin-Dependent Protein Kinase (CaMK), enhancing HIF-1α transcriptional activity. Prolonged VEGF stimulation leads to the scarring of tendons and tissues, resulting in ECM alteration and excessive collagen accumulation [128]. This is a similar etiology related to tendinopathy and AC; however, the mechanisms of hypoxia and AC are still unexplored.

Intracellular calcium is crucial for mitochondrial function and metabolism. The mitochondria, in turn, can produce reactive oxygen species (ROS) under hypoxic conditions. ROS can stabilize HIF-1α by inhibiting prolyl hydroxylase enzymes (PHDs) [129], which normally mark HIF-1α for degradation under normoxic (normal oxygen) conditions. Prolyl hydroxylase domains (PHDs), the enzymes responsible for hydroxylating and thereby marking HIF-1α for proteasomal degradation, are influenced by calcium levels. Changes in calcium concentration can affect these enzymes’ activity, indirectly impacting HIF-1α stability.

The endoplasmic reticulum (ER) and other calcium stores in the cell can release calcium in response to hypoxic stress [130,131,132]. The released calcium can activate a range of downstream signaling pathways that may lead to the stabilization of HIF-1α and the activation of HIF target genes.

Through these mechanisms, calcium can directly and indirectly affect HIF-1α stabilization, nuclear translocation, and transcriptional activity, ultimately influencing the cellular adaptive response to hypoxia.

## 9. Concluding Remarks and Perspective

In concluding the discussion on the interplay between calcium imbalance, the calcific tendinopathy of rotator cuffs, and adhesive capsulitis concerning metabolic mechanisms, it is evident that the intricate relationship between these conditions can significantly impact patient outcomes and management strategies.

Calcium homeostasis is crucial for numerous physiological processes, including muscle contraction and nerve transmission. An imbalance in calcium levels can lead to pathological conditions affecting musculoskeletal health. Calcific tendinopathy, characterized by tendon degeneration, pain, and impaired function, may be exacerbated by dysregulated calcium signaling, which contributes to altered cellular activities and extracellular matrix degradation.

Similarly, metabolic dysfunctions, including calcium imbalance, can influence the inflammatory and fibrotic processes central to adhesive capsulitis pathogenesis. The metabolic mechanisms underlying these conditions suggest that calcium imbalance can disrupt normal tendon and joint physiology through oxidative stress, inflammation, and changes in cellular apoptosis and proliferation rates. This emphasizes the importance of targeting metabolic pathways and maintaining calcium balance in therapeutic approaches.

Future research should focus on elucidating specific metabolic pathways and calcium-regulating factors involved in tendinopathy and adhesive capsulitis. This knowledge can inform the development of more effective interventions aimed at correcting calcium imbalance and restoring tissue homeostasis. In summary, understanding the interplay between calcium imbalance, calcific tendinopathy, and adhesive capsulitis highlights the importance of metabolic mechanisms in the pathology of these conditions. Clinicians and researchers should consider these interactions when devising comprehensive treatment plans and clinical trials to improve patient outcomes.

## Figures and Tables

**Table 1:** Pathophysiological link between calcific tendinopathy and shoulder arthrofibrosis (adhesive capsulitis).

Pathophysiological Link	Calcific Tendinopathy	Adhesive Capsulitis	Mechanisms/Implications
Chronic Micro-Leakage of Calcium	Calcium moves from tendon fibers into the joint’s synovial recesses, leading to inflammation	Inflammatory response in the shoulder capsule	This chronic leakage can initiate synovial inflammation, linking both conditions
Chemical Synovitis	Calcium deposits trigger a chemical inflammatory response	Results in pain and stiffness in the shoulder	Chemical irritation from calcium can exacerbate capsular inflammation
Intra-Articular Calcium Debris	Calcium debris from the rotator cuff tends to diffuse into the joint space during procedures	Associated with higher postoperative adhesive capsulitis risk	Arthroscopic interventions can inadvertently introduce calcium debris, promoting inflammation
Prolonged Pain-Induced Hypomobility	Chronic pain leads to decreased shoulder mobility	Results in fibrosis and further stiffness of the shoulder capsule	Limited movement due to pain can worsen both conditions
Inflammatory Response	Activation of immune pathways due to calcium accumulation	Inflammatory processes in the shoulder capsule	Shared inflammatory mechanisms can link the two conditions
Synovial/Capsular Inflammation	Initiated by the presence of calcium in the joint	Characterized by thickening and loss of mobility	Inflammation in the synovial tissue may lead to adhesive capsulitis

**Table 2:** Interconnected pathways and mechanisms influencing muscle regeneration, fibrosis, and calcium homeostasis.

Pathway/Component	Role/Function	Mechanisms/Interactions	Implications
Fibro-Adipogenic Progenitors (FAPs)	Muscle-resident interstitial cells with mesenchymal stem cell properties; essential for muscle homeostasis and regeneration	Differentiate into activated fibroblasts, adipocytes, and osteogenic cells, providing signals for muscle stem cell (MuSC) expansion and myogenesis	Dysregulation can lead to fibrosis and impaired muscle regeneration
Calcium Signaling	Critical for various cellular processes, including muscle contraction and cell growth	Calmodulin binds calcium, modulating activity of kinases and phosphatases; influences TGF-β signaling pathways	Calcium overload can lead to fibrosis and adipogenesis
TGF-β Signaling	Plays a key role in regulating muscle regeneration and fibrosis	Activates SMAD2, SMAD3 (canonical pathways) and PI3K-AKT, p38 MAPK (non-canonical pathways)	Dysregulation can lead to excessive fibrosis and impaired muscle repair
Calmodulin	Calcium-binding protein that mediates calcium signaling	Binds Ca^2+^, interacts with SMAD proteins, influences transcriptional activity of TGF-β	Overexpression can inhibit TGF-β effects, impacting fibrosis
IP3 Pathway	Mediates calcium release from intracellular stores	Activation of phospholipase C leads to IP3 generation, triggering calcium release and influx	Critical for TGF-β-induced calcium signaling
Myostatin (GDF8)	Regulates muscle growth and differentiation	Influences FAP activation via p38 MAPK and AKT pathways	Involved in various muscular disorders and metabolic conditions
Bone Morphogenetic Proteins (BMPs)	Induce osteogenic differentiation in mesenchymal stem cells (MSCs)	Bind to serine–threonine kinase receptors, activating SMAD and non-SMAD pathways	Promote bone formation and interact with calcium signaling
Inflammatory Cytokines (IL-6, IL-1)	Mediate inflammatory responses, linked to fibrosis and musculoskeletal disorders	Secreted by FAPs and osteoblasts, influencing MAPK signaling for skeletogenesis	Associated with the progression of rotator cuff diseases
Calcium Homeostasis	Maintained by parathyroid hormone (PTH) and calcitonin	PTH elevates plasma calcium levels by stimulating osteoclast activity; calcitonin decreases calcium levels	Imbalances can affect bone and muscle health
Calcium Dynamics in MSCs	Essential for osteogenic differentiation	Calcium and phosphate ions activate BMP/SMAD and RAS signaling pathways	Dysregulation can affect bone remodeling and contribute to disorder

## Abbreviations

AC	Adhesive capsulitis
BMI	Body mass index
BMP	Bone morphogenetic protein
COL1A1	Alpha1 chain of type I collagen
CRYab	Crystallin alpha B
DHPR	Dihydropyridine receptors
ECC	Excitation coupling contraction
ECM	Extracellular matrix
ERK	Extracellular signal regulated kinase
ER	Estrogen receptor
FAPs	Fibro-adipogenic progenitors
GSK3β	Glycogen synthase kinase 3
HIF	Hypoxia inducible factors
IGF	Insulin-like growth factor
IL	Interleukin
IP3	Inositol 1,4,5 trisphosphate
MAPK	Mitogen activated protein kinases
MMP	Matrix metalloproteinase
MSCs	Mesenchymal stem cells
MuSC	Muscle stem cell
NFAT	Nuclear factor of activated T cells
OSX	Osterix
PDGFRα	Platelet-derived growth factor receptor alpha isoform
PHDs	Prolyl hydroxylase enzymes
PI3K	Phosphatidylinositol 3 kinase
PTGES	Prostaglandin E Synthase
RCCT	Rotator cuff calcific tendinopathy
RCT	Rotator cuff tears
ROM	Range of motion
ROS	Reactive oxygen species
RUNX2	Runt-related transcriptional factor 2
RyR	Ryanodine receptors
SCA	Stem cell antigen
SCN4A	Sodium channels alpha subunit
SCX	Scleraxis
SERCA	Sarcoplasmic reticulum calcium ATPase pump
SMAD	Suppressor of mothers against decapentaplegic
TGFβ	Transforming growth factor β
TNMD	Tenomodulin
TSCs	Tendon stem cells
VEGF	Vascular endothelial growth factor

## References

[1] Chianca V., Albano D., Messina C., Midiri F., Mauri G., Aliprandi A., Catapano M., Pescatori L.C., Monaco C.G., Gitto S. (2018). Rotator Cuff Calcific Tendinopathy: From Diagnosis to Treatment. Acta Bio Medica Atenei Parm..

[2] Akbar M., Crowe L.A.N., McLean M., Garcia-Melchor E., MacDonald L., Carter K., Fazzi U.G., Martin D., Arthur A., Reilly J.H. (2021). Translational Targeting of Inflammation and Fibrosis in Frozen Shoulder: Molecular Dissection of the T Cell/IL-17A Axis. Proc. Natl. Acad. Sci. USA.

[3] Alghamdi A., Alyami A.H., Althaqafi R.M.M., Alzeyadi A., Alrubaei F.S., Alyami A.A., Singer M.S., Saati A.A., Alotaibi W.T., Alsharif M.O. (2023). Cytokines’ Role in the Pathogenesis and Their Targeting for the Prevention of Frozen Shoulder: A Narrative Review. Cureus.

[4] Brindisino F., Girardi G., Crestani M., Fiore A., Giovannico G., Garzonio F., Venturin D., Struyf F. (2023). Effectiveness of Electrophysical Agents in Subjects with Frozen Shoulder: A Systematic Review and Meta-Analysis. Disabil. Rehabil..

[5] Cao W., Chen J., Pu J., Fan Y., Cao Y. (2022). Risk Factors for the Onset of Frozen Shoulder in Middle-Aged and Elderly Subjects Within 1 Year of Discharge From a Hospitalization That Involved Intravenous Infusion: A Prospective Cohort Study. Front. Med..

[6] Chen J., Zhu J., Zhu T., Cui J., Deng Z., Chen K., Chang C., Geng Y., Chen F., Ouyang K. (2021). Pathological Changes of Frozen Shoulder in Rat Model and the Therapeutic Effect of PPAR-γ Agonist. J. Orthop. Res..

[7] Cher J.Z.B., Akbar M., Kitson S., Crowe L.A.N., Garcia-Melchor E., Hannah S.C., McLean M., Fazzi U.G., Kerr S.C., Murrell G.A.C. (2018). Alarmins in Frozen Shoulder: A Molecular Association Between Inflammation and Pain. Am. J. Sports Med..

[8] Cho C.-H., Song K.-S., Kim B.-S., Kim D.H., Lho Y.-M. (2018). Biological Aspect of Pathophysiology for Frozen Shoulder. BioMed Res. Int..

[9] De La Serna D., Navarro-Ledesma S., Alayón F., López E., Pruimboom L. (2021). A Comprehensive View of Frozen Shoulder: A Mystery Syndrome. Front. Med..

[10] Erickson B.J., Shishani Y., Bishop M.E., Romeo A.A., Gobezie R. (2019). Adhesive Capsulitis: Demographics and Predictive Factors for Success Following Steroid Injections and Surgical Intervention. Arthrosc. Sports Med. Rehabil..

[11] Kingston K., Curry E.J., Galvin J.W., Li X. (2018). Shoulder Adhesive Capsulitis: Epidemiology and Predictors of Surgery. J. Shoulder Elb. Surg..

[12] Kraal T., Lübbers J., Van Den Bekerom M.P.J., Alessie J., Van Kooyk Y., Eygendaal D., Koorevaar R.C.T. (2020). The Puzzling Pathophysiology of Frozen Shoulders—A Scoping Review. J. Exp. Orthop..

[13] Nagy M.T., MacFarlane R.J., Khan Y., Waseem M. (2013). The Frozen Shoulder: Myths and Realities. Open Orthop. J..

[14] Sun G., Li Q., Yin Y., Fu W., He K., Pen X. (2024). Risk Factors and Predictive Models for Frozen Shoulder. Sci. Rep..

[15] Merolla G., Bhat M.G., Paladini P., Porcellini G. (2015). Complications of Calcific Tendinitis of the Shoulder: A Concise Review. J. Orthop. Traumatol. Off. J. Ital. Soc. Orthop. Traumatol..

[16] Phansopkar P., Qureshi M.I. (2022). A Review on Current Notion in Frozen Shoulder: A Mystery Shoulder. Cureus.

[17] Millar N.L., Meakins A., Struyf F., Willmore E., Campbell A.L., Kirwan P.D., Akbar M., Moore L., Ronquillo J.C., Murrell G.A.C. (2022). Frozen Shoulder. Nat. Rev. Dis. Primer.

[18] Kelley M.J., Shaffer M.A., Kuhn J.E., Michener L.A., Seitz A.L., Uhl T.L., Godges J.J., McClure P. (2013). Shoulder Pain and Mobility Deficits: Adhesive Capsulitis: Clinical Practice Guidelines Linked to the International Classification of Functioning, Disability, and Health From the Orthopaedic Section of the American Physical Therapy Association. J. Orthop. Sports Phys. Ther..

[19] Hagiwara Y., Ando A., Onoda Y., Takemura T., Minowa T., Hanagata N., Tsuchiya M., Watanabe T., Chimoto E., Suda H. (2012). Coexistence of Fibrotic and Chondrogenic Process in the Capsule of Idiopathic Frozen Shoulders. Osteoarthr. Cartil..

[20] Le H.V., Lee S.J., Nazarian A., Rodriguez E.K. (2017). Adhesive Capsulitis of the Shoulder: Review of Pathophysiology and Current Clinical Treatments. Shoulder Elb..

[21] Zappia M., Reginelli A., Russo A., D’Agosto G.F., Di Pietto F., Genovese E.A., Coppolino F., Brunese L. (2013). Long Head of the Biceps Tendon and Rotator Interval. Musculoskelet. Surg..

[22] Draghi F., Scudeller L., Draghi A.G., Bortolotto C. (2015). Prevalence of Subacromial-Subdeltoid Bursitis in Shoulder Pain: An Ultrasonographic Study. J. Ultrasound.

[23] Picasso R., Pistoia F., Zaottini F., Marcenaro G., Miguel-Pérez M., Tagliafico A.S., Martinoli C. (2023). Adhesive Capsulitis of the Shoulder: Current Concepts on the Diagnostic Work-Up and Evidence-Based Protocol for Radiological Evaluation. Diagnostics.

[24] Hand C., Clipsham K., Rees J.L., Carr A.J. (2008). Long-Term Outcome of Frozen Shoulder. J. Shoulder Elb. Surg..

[25] Sansone V., Maiorano E., Galluzzo A., Pascale V. (2018). Calcific Tendinopathy of the Shoulder: Clinical Perspectives into the Mechanisms, Pathogenesis, and Treatment. Orthop. Res. Rev..

[26] Della Valle V., Bassi E.M., Calliada F. (2015). Migration of Calcium Deposits into Subacromial–Subdeltoid Bursa and into Humeral Head as a Rare Complication of Calcifying Tendinitis: Sonography and Imaging. J. Ultrasound.

[27] Kim M.-S., Kim I.-W., Lee S., Shin S.-J. (2020). Diagnosis and Treatment of Calcific Tendinitis of the Shoulder. Clin. Shoulder Elb..

[28] Becciolini M., Bonacchi G., Galletti S. (2016). Intramuscular Migration of Calcific Tendinopathy in the Rotator Cuff: Ultrasound Appearance and a Review of the Literature. J. Ultrasound.

[29] Loew M., Schnetzke M., Lichtenberg S. (2021). Current Treatment Concepts of Calcifying Tendinitis of the Shoulder: A Systematic Review. Obere Extrem..

[30] Spinnato P., Masuzzo O., Tuè G., Tucci F., Bevere A., Vita F., Cavallo M., Marinelli A., Miceli M. (2023). A Novel Ultrasound-Guided Interventional Procedure for the Combined Treatment of Rotator Cuff Calcific Tendinopathy Complicated with Adhesive Capsulitis: The “Rizzoli” Technique. Acad. Radiol..

[31] Furuhata R., Matsumura N., Yoshiyama A., Kamata Y., Takahashi M., Morioka H. (2020). Seasonal Variation in the Onset of Acute Calcific Tendinitis of Rotator Cuff. BMC Musculoskelet. Disord..

[32] Ricci V., Mezian K., Chang K.-V., Özçakar L. (2022). Clinical/Sonographic Assessment and Management of Calcific Tendinopathy of the Shoulder: A Narrative Review. Diagnostics.

[33] Cho C.-H., Bae K.-C., Kim D.-H. (2019). Treatment Strategy for Frozen Shoulder. Clin. Orthop. Surg..

[34] Hyatt H.W., Powers S.K. (2020). Disturbances in Calcium Homeostasis Promotes Skeletal Muscle Atrophy: Lessons From Ventilator-Induced Diaphragm Wasting. Front. Physiol..

[35] Sun X., Wang W., Dong Y., Wang Y., Zhang M., Wang Z., Yu X., Huang J., Cai H. (2020). Relationship between Calcium Circulation-Related Factors and Muscle Strength in Rat Sciatic Nerve Injury Model. Iran. J. Basic Med. Sci..

[36] Jayasinghe I.D., Munro M., Baddeley D., Launikonis B.S., Soeller C. (2014). Observation of the Molecular Organization of Calcium Release Sites in Fast- and Slow-Twitch Skeletal Muscle with Nanoscale Imaging. J. R. Soc. Interface.

[37] Mukund K., Subramaniam S. (2020). Skeletal Muscle: A Review of Molecular Structure and Function, in Health and Disease. WIREs Syst. Biol. Med..

[38] Berridge M.J., Bootman M.D., Roderick H.L. (2003). Calcium Signalling: Dynamics, Homeostasis and Remodelling. Nat. Rev. Mol. Cell Biol..

[39] Saran S., Babhulkar J.A., Gupta H., Chari B. (2024). Imaging of Calcific Tendinopathy: Natural History, Migration Patterns, Pitfalls, and Management: A Review. Br. J. Radiol..

[40] Lee K.J., Clegg P.D., Comerford E.J., Canty-Laird E.G. (2018). A Comparison of the Stem Cell Characteristics of Murine Tenocytes and Tendon-Derived Stem Cells. BMC Musculoskelet. Disord..

[41] Tuè G., Masuzzo O., Tucci F., Cavallo M., Parmeggiani A., Vita F., Patti A., Donati D., Marinelli A., Miceli M. (2024). Can Secondary Adhesive Capsulitis Complicate Calcific Tendinitis of the Rotator Cuff? An Ultrasound Imaging Analysis. Clin. Pract..

[42] Meißner J.D., Kubis H., Scheibe R.J., Gros G. (2000). Reversible Ca^2+^-induced Fast-to-slow Transition in Primary Skeletal Muscle Culture Cells at the mRNA Level. J. Physiol..

[43] Zhang C., Zhu J., Zhou Y., Thampatty B.P., Wang J.H.-C. (2019). Tendon Stem/Progenitor Cells and Their Interactions with Extracellular Matrix and Mechanical Loading. Stem Cells Int..

[44] Bi Y., Ehirchiou D., Kilts T.M., Inkson C.A., Embree M.C., Sonoyama W., Li L., Leet A.I., Seo B.-M., Zhang L. (2007). Identification of Tendon Stem/Progenitor Cells and the Role of the Extracellular Matrix in Their Niche. Nat. Med..

[45] Millar N.L., Reilly J.H., Kerr S.C., Campbell A.L., Little K.J., Leach W.J., Rooney B.P., Murrell G.A.C., McInnes I.B. (2012). Hypoxia: A Critical Regulator of Early Human Tendinopathy. Ann. Rheum. Dis..

[46] Steinmann S., Pfeifer C.G., Brochhausen C., Docheva D. (2020). Spectrum of Tendon Pathologies: Triggers, Trails and End-State. Int. J. Mol. Sci..

[47] Rui Y., Chan L., Chan K., Fu S., Gang L. (2011). Does Erroneous Differentiation of Tendon-Derived Stem Cells Contribute to the Pathogenesis of Calcifying Tendinopathy?. Chin. Med. J..

[48] Robinson D.M., Schowalter S., McInnis K.C. (2021). Update on Evaluation and Management of Calcific Tendinopathy. Curr. Phys. Med. Rehabil. Rep..

[49] Chen J., Jiang C., Yin L., Liu Y., He Y., Li S., Shen H. (2023). A Review of the Role of Tendon Stem Cells in Tendon-Bone Regeneration. Med. Sci. Monit..

[50] Passini F.S., Jaeger P.K., Saab A.S., Hanlon S., Chittim N.A., Arlt M.J., Ferrari K.D., Haenni D., Caprara S., Bollhalder M. (2021). Shear-Stress Sensing by PIEZO1 Regulates Tendon Stiffness in Rodents and Influences Jumping Performance in Humans. Nat. Biomed. Eng..

[51] Wunderli S.L., Widmer J., Amrein N., Foolen J., Silvan U., Leupin O., Snedeker J.G. (2018). Minimal Mechanical Load and Tissue Culture Conditions Preserve Native Cell Phenotype and Morphology in Tendon—A Novel Ex Vivo Mouse Explant Model. J. Orthop. Res..

[52] Tohidnezhad M., Zander J., Slowik A., Kubo Y., Dursun G., Willenberg W., Zendedel A., Kweider N., Stoffel M., Pufe T. (2020). Impact of Uniaxial Stretching on Both Gliding and Traction Areas of Tendon Explants in a Novel Bioreactor. Int. J. Mol. Sci..

[53] Maeda E., Shelton J.C., Bader D.L., Lee D.A. (2007). Time Dependence of Cyclic Tensile Strain on Collagen Production in Tendon Fascicles. Biochem. Biophys. Res. Commun..

[54] Maeda E., Shelton J.C., Bader D.L., Lee D.A. (2009). Differential Regulation of Gene Expression in Isolated Tendon Fascicles Exposed to Cyclic Tensile Strain in Vitro. J. Appl. Physiol..

[55] Devkota A.C., Tsuzaki M., Almekinders L.C., Banes A.J., Weinhold P.S. (2007). Distributing a Fixed Amount of Cyclic Loading to Tendon Explants over Longer Periods Induces Greater Cellular and Mechanical Responses. J. Orthop. Res..

[56] Wang T., Lin Z., Ni M., Thien C., Day R.E., Gardiner B., Rubenson J., Kirk T.B., Smith D.W., Wang A. (2015). Cyclic Mechanical Stimulation Rescues Achilles Tendon from Degeneration in a Bioreactor System. J. Orthop. Res..

[57] Screen H.R., Shelton J.C., Bader D.L., Lee D.A. (2005). Cyclic Tensile Strain Upregulates Collagen Synthesis in Isolated Tendon Fascicles. Biochem. Biophys. Res. Commun..

[58] Legerlotz K., Jones G., Screen H., Riley G. (2013). Cyclic Loading of Tendon Fascicles Using a Novel Fatigue Loading System Increases Interleukin-6 Expression by Tenocytes. Scand. J. Med. Sci. Sports.

[59] Szczesny S.E., Aeppli C., David A., Mauck R.L. (2018). Fatigue Loading of Tendon Results in Collagen Kinking and Denaturation but Does Not Change Local Tissue Mechanics. J. Biomech..

[60] Cho C.-H., Lho Y.-M., Hwang I., Kim D.H. (2019). Role of Matrix Metalloproteinases 2 and 9 in the Development of Frozen Shoulder: Human Data and Experimental Analysis in a Rat Contracture Model. J. Shoulder Elb. Surg..

[61] Wu S.Y., Kim W., Kremen T.J. (2022). In Vitro Cellular Strain Models of Tendon Biology and Tenogenic Differentiation. Front. Bioeng. Biotechnol..

[62] Tang H., Zeng R., He E., Zhang I., Ding C., Zhang A. (2022). Piezo-Type Mechanosensitive Ion Channel Component 1 (Piezo1): A Promising Therapeutic Target and Its Modulators: Miniperspective. J. Med. Chem..

[63] Szabó L., Balogh N., Tóth A., Angyal Á., Gönczi M., Csiki D.M., Tóth C., Balatoni I., Jeney V., Csernoch L. (2022). The Mechanosensitive Piezo1 Channels Contribute to the Arterial Medial Calcification. Front. Physiol..

[64] Thien N.D., Hai-Nam N., Anh D.T., Baecker D. (2024). Piezo1 and Its Inhibitors: Overview and Perspectives. Eur. J. Med. Chem..

[65] Yan Y., Zhou M., Meng K., Zhou C., Jia X., Li X., Cui D., Yu M., Tang Y., Li M. (2023). Salvianolic Acid B Attenuates Inflammation and Prevent Pathologic Fibrosis by Inhibiting CD36-Mediated Activation of the PI3K-Akt Signaling Pathway in Frozen Shoulder. Front. Pharmacol..

[66] Yan Y., Li X., Chen C., Cui D., Wang Z., Li M., Long Y., Zhang J., Li C., Wang Z. (2024). A Mussel-Inspired, Antibacterial, Antioxidant, Injectable Composite Hydrogel for the Sustain Delivery of Salvianolic Acid B for the Treatment of Frozen Shoulder. Bioact. Mater..

[67] Cao C., Ren Y., Barnett A.S., Mirando A.J., Rouse D., Mun S.H., Park-Min K.-H., McNulty A.L., Guilak F., Karner C.M. (2017). Increased Ca^2+^ Signaling through Ca_V_1. 2 Promotes Bone Formation and Prevents Estrogen Deficiency–Induced Bone Loss. JCI Insight.

[68] Cao C., Oswald A.B., Fabella B.A., Ren Y., Rodriguiz R., Trainor G., Greenblatt M.B., Hilton M.J., Pitt G.S. (2019). The CaV1. 2 L-Type Calcium Channel Regulates Bone Homeostasis in the Middle and Inner Ear. Bone.

[69] Ramachandran K.V., Hennessey J.A., Barnett A.S., Yin X., Stadt H.A., Foster E., Shah R.A., Yazawa M., Dolmetsch R.E., Kirby M.L. (2013). Calcium Influx through L-Type Ca_V_1.2 Ca^2+^ Channels Regulates Mandibular Development. J. Clin. Investig..

[70] Li H., Korcari A., Ciufo D., Mendias C.L., Rodeo S.A., Buckley M.R., Loiselle A.E., Pitt G.S., Cao C. (2023). Increased Ca^2+^ Signaling through Ca_V_1.2 Induces Tendon Hypertrophy with Increased Collagen Fibrillogenesis and Biomechanical Properties. FASEB J..

[71] Ross S.E., Hemati N., Longo K.A., Bennett C.N., Lucas P.C., Erickson R.L., MacDougald O.A. (2000). Inhibition of Adipogenesis by Wnt Signaling. Science.

[72] Mueller A.A., Van Velthoven C.T., Fukumoto K.D., Cheung T.H., Rando T.A. (2016). Intronic Polyadenylation of PDGFRα in Resident Stem Cells Attenuates Muscle Fibrosis. Nature.

[73] Giuliani G., Rosina M., Reggio A. (2022). Signaling Pathways Regulating the Fate of Fibro/Adipogenic Progenitors (FAPs) in Skeletal Muscle Regeneration and Disease. FEBS J..

[74] Kharraz Y., Guerra J., Pessina P., Serrano A.L., Muñoz-Cánoves P. (2014). Understanding the Process of Fibrosis in Duchenne Muscular Dystrophy. BioMed Res. Int..

[75] Joe A.W., Yi L., Natarajan A., Le Grand F., So L., Wang J., Rudnicki M.A., Rossi F.M. (2010). Muscle Injury Activates Resident Fibro/Adipogenic Progenitors That Facilitate Myogenesis. Nat. Cell Biol..

[76] Uezumi A., Fukada S., Yamamoto N., Takeda S., Tsuchida K. (2010). Mesenchymal Progenitors Distinct from Satellite Cells Contribute to Ectopic Fat Cell Formation in Skeletal Muscle. Nat. Cell Biol..

[77] Pessina P., Cabrera D., Morales M.G., Riquelme C.A., Gutiérrez J., Serrano A.L., Brandan E., Muñoz-Cánoves P. (2014). Novel and Optimized Strategies for Inducing Fibrosis in Vivo: Focus on Duchenne Muscular Dystrophy. Skelet. Muscle.

[78] Martin A.B., Cardenas M.A., Andersen R.K., Bowman A.I., Hillier E.A., Bensmaia S., Fuglevand A.J., Gothard K.M. (2023). A Context-Dependent Switch from Sensing to Feeling in the Primate Amygdala. Cell Rep..

[79] Yang R., Tang Y., Hou J., Yu M., Long Y., Yamuhanmode A., Li Q., Li F., Zhang Y., Warsame M. (2022). Fibrosis in Frozen Shoulder: Activation of IL-6 through PI3K-Akt Signaling Pathway in Synovial Fibroblast. Mol. Immunol..

[80] Bonnieu A., Carnac G., Vernus B. (2007). Myostatin in the Pathophysiology of Skeletal Muscle. Curr. Genom..

[81] Yang M., Liu C., Jiang N., Liu Y., Luo S., Li C., Zhao H., Han Y., Chen W., Li L. (2023). Myostatin: A Potential Therapeutic Target for Metabolic Syndrome. Front. Endocrinol..

[82] Dong J., Dong Y., Chen Z., Mitch W.E., Zhang L. (2017). The Pathway to Muscle Fibrosis Depends on Myostatin Stimulating the Differentiation of Fibro/Adipogenic Progenitor Cells in Chronic Kidney Disease. Kidney Int..

[83] Lee S.-J., Bhasin S., Klickstein L., Krishnan V., Rooks D. (2023). Challenges and Future Prospects of Targeting Myostatin/Activin A Signaling to Treat Diseases of Muscle Loss and Metabolic Dysfunction. J. Gerontol. Ser. A.

[84] McGowan T.A., Madesh M., Zhu Y., Wang L., Russo M., Deelman L., Henning R., Joseph S., Hajnoczky G., Sharma K. (2002). TGF-β-Induced Ca^2+^ Influx Involves the Type III IP_3_ Receptor and Regulates Actin Cytoskeleton. Am. J. Physiol. Ren. Physiol..

[85] Wicks S.J., Lui S., Abdel-Wahab N., Mason R.M., Chantry A. (2000). Inactivation of Smad-Transforming Growth Factor β Signaling by Ca2+-Calmodulin-Dependent Protein Kinase II. Mol. Cell. Biol..

[86] Pacher P., Sharma K., Csordás G., Zhu Y., Hajnóczky G. (2008). Uncoupling of ER-Mitochondrial Calcium Communication by Transforming Growth Factor-β. Am. J. Physiol. Ren. Physiol..

[87] Deng Z., Fan T., Xiao C., Tian H., Zheng Y., Li C., He J. (2024). TGF-β Signaling in Health, Disease and Therapeutics. Signal Transduct. Target. Ther..

[88] Song Z., Wang Y., Zhang F., Yao F., Sun C. (2019). Calcium Signaling Pathways: Key Pathways in the Regulation of Obesity. Int. J. Mol. Sci..

[89] Coultrap S.J., Buard I., Kulbe J.R., Dell’Acqua M.L., Bayer K.U. (2010). CaMKII Autonomy Is Substrate-Dependent and Further Stimulated by Ca^2+^/Calmodulin. J. Biol. Chem..

[90] Stratton M.M., Chao L.H., Schulman H., Kuriyan J. (2013). Structural Studies on the Regulation of Ca^2+^/Calmodulin Dependent Protein Kinase II. Curr. Opin. Struct. Biol..

[91] Chao L.H., Stratton M.M., Lee I.-H., Rosenberg O.S., Levitz J., Mandell D.J., Kortemme T., Groves J.T., Schulman H., Kuriyan J. (2011). A Mechanism for Tunable Autoinhibition in the Structure of a Human Ca^2+^/Calmodulin- Dependent Kinase II Holoenzyme. Cell.

[92] Liu G., Li M., Xu Y., Wu S., Saeed M., Sun C. (2017). ColXV Promotes Adipocyte Differentiation via Inhibiting DNA Methylation and cAMP/PKA Pathway in Mice. Oncotarget.

[93] Wada N., Hashinaga T., Otabe S., Yuan X., Kurita Y., Kakino S., Ohoki T., Nakayama H., Fukutani T., Tajiri Y. (2013). Selective Modulation of Wnt Ligands and Their Receptors in Adipose Tissue by Chronic Hyperadiponectinemia. PLoS ONE.

[94] Benchoula K., Mediani A., Hwa W.E. (2023). The Functions of Ca^2+^/Calmodulin-Dependent Protein Kinase II (CaMKII) in Diabetes Progression. J. Cell Commun. Signal..

[95] Deng G., Wei Y. (2023). The Causal Relationship between Hypothyroidism and Frozen Shoulder: A Two-Sample Mendelian Randomization. Medicine.

[96] Carter P.H., Schipani E. (2006). The Roles of Parathyroid Hormone and Calcitonin in Bone Remodeling: Prospects for Novel Therapeutics. Endocr. Metab. Immune Disord. Drug Targets Former. Curr. Drug Targets Immune Endocr. Metab. Disord..

[97] Babić Leko M., Pleić N., Gunjača I., Zemunik T. (2021). Environmental Factors That Affect Parathyroid Hormone and Calcitonin Levels. Int. J. Mol. Sci..

[98] Rouhani A., Mardani-Kivi M., Bazavar M., Barzgar M., Tabrizi A., Hashemi-Motlagh K., Saheb-Ekhtiari K. (2016). Calcitonin Effects on Shoulder Adhesive Capsulitis. Eur. J. Orthop. Surg. Traumatol..

[99] Yang R., Deng H., Hou J., Li W., Zhang C., Yu M., Tang Y., Li Q., Li F., Song B. (2020). Investigation of Salmon Calcitonin in Regulating Fibrosis-related Molecule Production and Cell-substrate Adhesion in Frozen Shoulder Synovial/Capsular Fibroblasts. J. Orthop. Res..

[100] Longo U.G., Mazzola A., Carotti S., Francesconi M., Catapano S., Magrì F., Perrone G., Morini S., De Salvatore S., Denaro V. (2021). The Role of Estrogen and Progesterone Receptors in the Rotator Cuff Disease: A Retrospective Cohort Stud y. BMC Musculoskelet. Disord..

[101] Cogan C.J., Cevallos N., Freshman R.D., Lansdown D., Feeley B.T., Zhang A.L. (2022). Evaluating Utilization Trends in Adhesive Capsulitis of the Shoulder: A Retrospective Cohort Analysis of a Large Database. Orthop. J. Sports Med..

[102] Saltzman E., Kennedy J., Ford A., Reinke E., Green C., Poehlein E., Wittstein J. (2023). Poster 188: Is Hormone Replacing Therapy Associated with Reduced Risk of Adhesive Capsulitis in Menopausal Women? A Single Center Analysis. Orthop. J. Sports Med..

[103] Heldring N., Pike A., Andersson S., Matthews J., Cheng G., Hartman J., Tujague M., Ström A., Treuter E., Warner M. (2007). Estrogen Receptors: How Do They Signal and What Are Their Targets. Physiol. Rev..

[104] Granéli C., Thorfve A., Ruetschi U., Brisby H., Thomsen P., Lindahl A., Karlsson C. (2014). Novel Markers of Osteogenic and Adipogenic Differentiation of Human Bone Marrow Stromal Cells Identified Using a Quantitative Proteomics Approach. Stem Cell Res..

[105] Viti F., Landini M., Mezzelani A., Petecchia L., Milanesi L., Scaglione S. (2016). Osteogenic Differentiation of MSC through Calcium Signaling Activation: Transcriptomics and Functional Analysis. PLoS ONE.

[106] Barradas A.M., Fernandes H.A., Groen N., Chai Y.C., Schrooten J., van de Peppel J., Van Leeuwen J.P., Van Blitterswijk C.A., de Boer J. (2012). A Calcium-Induced Signaling Cascade Leading to Osteogenic Differentiation of Human Bone Marrow-Derived Mesenchymal Stromal Cells. Biomaterials.

[107] Regan J.N., Waning D.L., Guise T.A. (2016). Skeletal Muscle Ca^2+^ Mishandling: Another Effect of Bone-to-Muscle Signaling. Semin. Cell Dev. Biol..

[108] Zhu Y., Chen L., Song B., Cui Z., Chen G., Yu Z., Song B. (2022). Insulin-like Growth Factor-2 (IGF-2) in Fibrosis. Biomolecules.

[109] Monaco S., Illario M., Rusciano M.R., Gragnaniello G., Di Spigna G., Leggiero E., Pastore L., Fenzi G., Rossi G., Vitale M. (2009). Insulin Stimulates Fibroblast Proliferation through Calcium-Calmodulin-Dependent Kinase II. Cell Cycle.

[110] Aquino-Martínez R., Artigas N., Gámez B., Rosa J.L., Ventura F. (2017). Extracellular Calcium Promotes Bone Formation from Bone Marrow Mesenchymal Stem Cells by Amplifying the Effects of BMP-2 on SMAD Signalling. PLoS ONE.

[111] Zhang Y.E. (2009). Non-Smad Pathways in TGF-β Signaling. Cell Res..

[112] Rodríguez-Carballo E., Ulsamer A., Susperregui A.R., Manzanares-Céspedes C., Sánchez-García E., Bartrons R., Rosa J.L., Ventura F. (2011). Conserved Regulatory Motifs in Osteogenic Gene Promoters Integrate Cooperative Effects of Canonical Wnt and BMP Pathways. J. Bone Miner. Res..

[113] Gámez B., Rodríguez-Carballo E., Graupera M., Rosa J.L., Ventura F. (2016). Class I PI-3-kinase Signaling Is Critical for Bone Formation through Regulation of SMAD1 Activity in Osteoblasts. J. Bone Miner. Res..

[114] Mandal C.C., Das F., Ganapathy S., Harris S.E., Choudhury G.G., Ghosh-Choudhury N. (2016). Bone Morphogenetic Protein-2 (BMP-2) Activates NFATc1 Transcription Factor via an Autoregulatory Loop Involving Smad/Akt/Ca^2+^ Signaling. J. Biol. Chem..

[115] Koga T., Matsui Y., Asagiri M., Kodama T., de Crombrugghe B., Nakashima K., Takayanagi H. (2005). NFAT and Osterix Cooperatively Regulate Bone Formation. Nat. Med..

[116] Fromigué O., Haÿ E., Barbara A., Marie P.J. (2010). Essential Role of Nuclear Factor of Activated T Cells (NFAT)-Mediated Wnt Signaling in Osteoblast Differentiation Induced by Strontium Ranelate. J. Biol. Chem..

[117] Fuentealba L.C., Eivers E., Ikeda A., Hurtado C., Kuroda H., Pera E.M., De Robertis E.M. (2007). Integrating Patterning Signals: Wnt/GSK3 Regulates the Duration of the BMP/Smad1 Signal. Cell.

[118] Majumdar M.K., Thiede M.A., Haynesworth S.E., Bruder S.P., Gerson S.L. (2000). Human Marrow-Derived Mesenchymal Stem Cells (MSCs) Express Hematopoietic Cytokines and Support Long-Term Hematopoiesis When Differentiated toward Stromal and Osteogenic Lineages. J. Hematother. Stem Cell Res..

[119] Rezaee F., Rellick S.L., Piedimonte G., Akers S.M., O’Leary H.A., Martin K., Craig M.D., Gibson L.F. (2010). Neurotrophins Regulate Bone Marrow Stromal Cell IL-6 Expression through the MAPK Pathway. PLoS ONE.

[120] Lee M.W., Kim D.S., Ryu S., Jang I.K., Kim H.J., Yang J.M., Lee D.-H., Lee S.H., Son M.H., Cheuh H.W. (2013). Effect of Ex Vivo Culture Conditions on Immunosuppression by Human Mesenchymal Stem Cells. BioMed Res. Int..

[121] Ren J., Jin P., Sabatino M., Balakumaran A., Feng J., Kuznetsov S.A., Klein H.G., Robey P.G., Stroncek D.F. (2011). Global Transcriptome Analysis of Human Bone Marrow Stromal Cells (BMSC) Reveals Proliferative, Mobile and Interactive Cells That Produce Abundant Extracellular Matrix Proteins, Some of Which May Affect BMSC Potency. Cytotherapy.

[122] Deshpande S., James A.W., Blough J., Donneys A., Wang S.C., Cederna P.S., Buchman S.R., Levi B. (2013). Reconciling the Effects of Inflammatory Cytokines on Mesenchymal Cell Osteogenic Differentiation. J. Surg. Res..

[123] Feng Y., Yang C., Yang W., Jiang T. (2019). Effect of Dexamethasone on TGF-Β1/Smad3 Signalling Pathway in Airway Remodelling Model of Asthmatic Rats. J. Coll. Physicians Surg. Pak..

[124] Ecker Cohen O., Neuman S., Natan Y., Levy A., Blum Y.D., Amselem S., Bavli D., Ben Y. (2024). Amorphous Calcium Carbonate Enhances Osteogenic Differentiation and Myotube Formation of Human Bone Marrow Derived Mesenchymal Stem Cells and Primary Skeletal Muscle Cells under Microgravity Conditions. Life Sci. Space Res..

[125] Huston P. (2022). A Sedentary and Unhealthy Lifestyle Fuels Chronic Disease Progression by Changing Interstitial Cell Behaviour: A Network Analysis. Front. Physiol..

[126] Taylor C.T., Scholz C.C. (2022). The Effect of HIF on Metabolism and Immunity. Nat. Rev. Nephrol..

[127] Liu X., Zhu B., Li Y., Liu X., Guo S., Wang C., Li S., Wang D. (2021). The Role of Vascular Endothelial Growth Factor in Tendon Healing. Front. Physiol..

[128] Wilgus T.A. (2019). Vascular Endothelial Growth Factor and Cutaneous Scarring. Adv. Wound Care.

[129] Willson J.A., Arienti S., Sadiku P., Reyes L., Coelho P., Morrison T., Rinaldi G., Dockrell D.H., Whyte M.K.B., Walmsley S.R. (2022). Neutrophil HIF-1α Stabilization Is Augmented by Mitochondrial ROS Produced via the Glycerol 3-Phosphate Shuttle. Blood.

[130] Lee A., Derricks K., Minns M., Ji S., Chi C., Nugent M.A., Trinkaus-Randall V. (2014). Hypoxia-Induced Changes in Ca^2+^ Mobilization and Protein Phosphorylation Implicated in Impaired Wound Healing. Am. J. Physiol. Cell Physiol..

[131] Rossi A., Pizzo P., Filadi R. (2019). Calcium, Mitochondria and Cell Metabolism: A Functional Triangle in Bioenergetics. Biochim. Biophys. Acta BBA Mol. Cell Res..

[132] Seta K.A., Yuan Y., Spicer Z., Lu G., Bedard J., Ferguson T.K., Pathrose P., Cole-Strauss A., Kaufhold A., Millhorn D.E. (2004). The Role of Calcium in Hypoxia-Induced Signal Transduction and Gene Expression. Cell Calcium.

